# Surgical vs. catheter-based paravalvular mitral valve leak closure (trans apical approach). Early results. Single center experience.

**DOI:** 10.1186/1749-8090-10-S1-A320

**Published:** 2015-12-16

**Authors:** Aleksejus Zorinas, Vilius Janusauskas, Rokas Simkauskas, Kestutis Rucinskas, Audrius Aidietis

**Affiliations:** 1 Department of Cardiovascular Medicine, Vilnius University, Vilnius, LT-08661, Lithuania; 2 Faculty of Medicine, Vilnius University, Vilnius, LT-03101, Lithuania

## Background/Introduction

Following surgical mitral valve replacement paravalvular leaks may occur in up to 17% of patients. A significant fraction of these patients present with a symptoms of heart failure and/or anaemia. Conventional surgical closure is associated with increased morbidity and mortality. Alternative transcatheter closure has been developed and being introduced into the clinical practice with a reasonable success. More evidence is needed to compare the efficacy and safety between surgical and catheter-based paravalvular mitral valve leak closure.

## Aims/Objectives

To compare efficacy and safety between two treatment methods of mitral valve paravalvular leak closure.

## Method

A retrospective analysis of patients' medical records treated for mitral paravalvular leak at our institution in year 2005-2015. 41 patients had paravalvular leak closure. 31 patients had paravalvular leak repaired via conventional surgery, and 10 patients had catheter-based procedure (trans apical approach). Patients' data, operative variables, postoperative complications, 1 and 4 months postoperative results were analyzed.

## Results

Patients in a catheter-based paravalvular leak closure group were older (71 ± 6 years vs. 63 ± 8 years, p = 0,004), and had higher incidence of essential hypertension (8 (80%) vs. 10 (32,3%), p = 0,008). Procedure was longer in surgical closure group (270 ± 98 min vs171 ± 86 min, p = 0,007). Early after the treatment mild/moderate regurgitation of a paravalvular leak was found more frequently in a catheter based paravalvular leak closure group (5 (50%) vs. 1 with severe regurgitation in conventional surgery group (3,45%), p = 0,0004).

## Discussion/Conclusion

Catheter-based closure of a paravalvular leak is reserved for older and sicker patients. Although the procedure of catheter-based paravalvular leak closure is quicker, patients have higher incidence of mild/moderate paravalvular leak after the procedure, which has reduced over time. With more clinical experience and development of special equipment, catheter-based paravalvular leak closure could be a possible alternative to the conventional operation.

